# Subtype Specific Elevated Expression of Hyaluronidase-1 (HYAL-1) in Epithelial Ovarian Cancer

**DOI:** 10.1371/journal.pone.0020705

**Published:** 2011-06-10

**Authors:** Paule Héléna Yoffou, Lydia Edjekouane, Liliane Meunier, André Tremblay, Diane Michèle Provencher, Anne-Marie Mes-Masson, Euridice Carmona

**Affiliations:** 1 Maisonneuve-Rosemont Hospital Research Center, Montreal, Quebec, Canada; 2 Department of Medicine, University of Montreal, Montreal, Quebec, Canada; 3 Department of Obstetrics and Gynecology, University of Montreal, Montreal, Quebec, Canada; 4 Department of Biochemistry, University of Montreal, Montreal, Quebec, Canada; 5 Centre de recherche du Centre hospitalier de l'Université de Montréal/Institut du cancer de Montréal, Montreal, Quebec, Canada; 6 Research Center, Centre hospitalier universitaire Ste-Justine, Montreal, Quebec, Canada; University of Hong Kong, Hong Kong

## Abstract

**Background:**

Epithelial ovarian cancer (EOC) is morphologically heterogeneous being classified as serous, endometrioid, clear cell, or mucinous. Molecular genetic analysis has suggested a role for tumor suppressor genes located at chromosome 3p in serous EOC pathogenesis. Our objective was to evaluate the expression of *HYAL1*, located at chromosome 3p21.3, in these EOC subtypes, and to investigate its correlation with the expression of steroid hormone receptors.

**Methodology/Principal Findings:**

We determined the mRNA expression of *HYAL1*, estrogen receptor (ER)-α, ERβ and progesterone receptor (PR) in EOC tumor samples and cell lines using quantitative RT-PCR. We also examined the expression of these genes in a publicly available microarray dataset. HYAL-1 enzyme activity was measured in EOC cell lines and in plasma samples from patients. We found that *HYAL1* mRNA expression was elevated in clear cell and mucinous EOC tissue samples, but not in serous and endometrioid samples, normal ovaries or benign tumors. Similar results were obtained by two different techniques and with tissue sample cohorts from two independent institutions. Concordantly, *HYAL1* mRNA levels and enzymatic activity were elevated only in EOC cell lines derived from clear cell and mucinous subtypes. We also showed that *HYAL1* mRNA was inversely correlated to that of ERα specifically in clear cell and mucinous EOCs. Additionally, ectopic expression of ERα in a clear cell EOC cell line (ER- and PR-negative) induced 50% reduction of *HYAL1* mRNA expression, supporting a role of ERα in *HYAL1* gene regulation. Significantly, HYAL-1 activity was also high in the plasma of patients with these EOC subtypes.

**Conclusions/Significance:**

This is the first report showing high HYAL-1 levels in EOC and demonstrating *HYAL1* gene repression by ERα. Our results identify Hyaluronidase-1 as a potential target/biomarker for clear cell and mucinous EOCs and especially in tumors with low ERα levels.

## Introduction

Epithelial ovarian cancer (EOC) is the leading cause of death from gynecologic cancer in most Western countries [1]. Because of its asymptomatic growth and the lack of effective screening methods, about 70% of all cases are diagnosed in an advanced stage, with only modest improvements in survival over the past 40 years [1]. Although most patients respond to chemotherapy initially, recurrence rates are very high resulting in the general poor prognosis seen in these patients [2]. Furthermore, EOCs are morphologically heterogeneous, and different histopathological subtypes have distinct molecular characteristics and diverse response to treatment [3]. EOC can be classified as serous, endometrioid, clear cell or mucinous which correspond to the different types of epithelia present in the female reproductive tract [4]. Differences in chemotherapy response and patient outcomes probably result from the molecular heterogeneity of these morphologically distinct EOCs [3]. For instance, *TP53* mutations are frequently observed in serous and endometrioid cancers, but are scarcely detected in clear cell and mucinous EOCs [3]. It is also known that the frequency of chromosomal instability is higher in serous EOC than in the other subtypes [3].

In serous EOC, molecular genetic analysis has suggested a role for tumor suppressor genes located on the short arm of chromosome 3 (3p) in the pathogenesis of this disease [5]. Transcriptome analysis of chromosome 3 genes identified several differentially expressed genes in EOC cell lines and ovarian tumors when compared to normal ovarian surface epithelial (NOSE) cells [6], [7]. Chromosomal aberrations in 3p21.3 are frequently found in lung, renal and breast cancers, suggesting that they harbor tumor suppressor genes [8]. Located on chromosome 3p21.3 is a cluster of genes, named hyaluronidases (*HYAL1*, *HYAL2* and *HYAL3*), that are the most frequent target of homozygous deletions in lung cancer [8].

Mammalian hyaluronidases (EC 3.2.1.35) are endo-*N*-acetylhexosaminidases which hydrolyze the glycosaminoglycan hyaluronan. They comprise a family of 6–7 genes with approximately 40% identity among each other [9], [10]. In humans, they are clustered in groups of three on chromosomes 3p21.3 (*HYAL1*, *HYAL2* and *HYAL3*) and 7q31.3 (*HYAL4*, *PH20*/*SPAM1* and *HYALP1*), with *HYALP1* being a pseudogene and *HYAL4* coding for a chondroitinase enzyme [9], [11]. Therefore, in humans, there are four hyaluronidases, HYAL-1, -2, -3 and PH20/Spam1, the latter being mainly expressed in the male reproductive tract and having an important role in fertilization [12]. On the other hand, hyaluronidases located on chromosome 3 are ubiquitously expressed. HYAL-1 and HYAL-2 are the main somatic hyaluronidases responsible for hyaluronan turnover and are known to have several physiological and pathological roles [9], [13], such as wound healing, inflammation and osteoarthritis. In contrast, Hyal-3 has been described to be devoid of hyaluronan enzymatic activity [14] and its physiological role still remains to be determined.

In ovarian cancer, allelic imbalance of these three genes (*HYAL1*, *HYAL2* and *HYAL3*) has been shown in tumor and stroma tissues [15]. In particular, *HYAL-1* mRNA expression was found to be significantly reduced in serous EOC when compared to normal ovaries [16], while unchanged or a tendency for decreased HYAL-1 activity was reported in EOC tissue extracts [16], [17]. In accordance with this observation, extracellular accumulation of hyaluronan is often observed in ovarian tumor stroma and pericellular matrix, and is associated with poor disease outcome [17], [18]. In addition, several reports have demonstrated interactions between hyaluronan and membrane receptors, such as CD44, which promotes the association of CD44 with certain cytoskeletal proteins (e.g. ankyrin, RhoGTPases, Cdc42) generating specific signalling events promoting ovarian cancer cell adhesion, migration and survival [19].

In contrast, levels of both hyaluronan and HYAL-1 have been reported to be increased in bladder, prostate and head and neck cancers, and to be implicated in tumor progression and metastasis [20]–[22]. Interestingly, elevated extracellular hyaluronan is mainly found in tumor stroma while elevated HYAL-1 levels are detected in tumor tissues, suggesting a cross-talk between these two tissue types. High levels of HYAL-1 expression are also found in breast cancer and glioblastomas, and are correlated with metastatic tumors [22], [23]. Interestingly, estrogen receptor (ER) negative breast cancer cell lines, which tend to be more aggressive, have enhanced hyaluronidase activity when compared to ER positive cell lines [24]. By a mechanism yet unknown, HYAL-1 induces cell cycle transition and up-regulates the levels of positive regulators of G2-M transition (e.g. cdc25c, cyclin B1, cdk10) in bladder, prostate and oral squamous cancer cell lines [25]–[27]. HYAL-1 also enhances angiogenesis, probably by generating hyaluronan fragments of distinct sizes which possess the ability to stimulate endothelial cell proliferation and capillary formation [28].

Therefore, levels of hyaluronidase expression may vary depending on tumor type and on its aggressive behaviour. In the present work we performed a detailed study on the expression of HYAL-1 in ovarian cancer tissue samples representing four different histopathological subtypes and showed elevated levels of this enzyme in clear cell and mucinous EOCs, but not in serous or endometrioid. We also demonstrated that levels of *HYAL1* mRNA in clear cell and mucinous EOCs were inversely correlated with those of ERα. Significantly, we showed that ectopic expression of ERα induced a 50% decrease in *HYAL1* mRNA expression in a clear cell EOC cell line. To our knowledge, this is the first report: i) demonstrating increased HYAL-1 expression in specific morphological EOC subtypes, ii) showing an inverse correlation between *HYAL1* and steroid hormones in tissue samples, and iii) implicating *HYAL1* gene as an ERα target for gene repression. Finally, we showed a 2.1–2.8 fold increase in the plasma levels of this enzyme in patients with clear cell and mucinous EOC, but not in those with serous or endometrioid tumors, when compared to patients with benign cysts. Our present results identify HYAL-1 as a potential biomarker for the detection of these two distinct EOC subtypes.

## Materials and Methods

### Clinical samples

Tissue tumor samples and EDTA-collected plasma were obtained, with informed consent, from participants undergoing surgeries performed at the Centre hospitalier de l'Université de Montréal (CHUM) Hôpital Notre-Dame. The policies for collection and use of tissue and blood samples were approved by the Research Ethics Committee of the CHUM. Only tumors from chemotherapy naïve patients were used. Histopathology, grade and stage of tumors were assigned according to the International Federation of Gynecology and Obstetrics (FIGO) criteria. As normal ovary has little epithelial content, benign serous ovarian tumors were used as a control. Information concerning the samples used in the present study is summarized in Table 1.

**Table 1:** Samples and clinical data of ovarian cancer patients for each analysis dataset.

*Dataset*	*Samples*		*G1*			*G2*			*G3*		
		*Stage*	*1–2*	*3–4*	*n.a*	*1–2*	*3–4*	*n.a*	*1–2*	*3–4*	*n.a*
Q-PCR(n = 53)	Benign (n = 15) :serous-subtype (14)mucinous-subtype (1)										
	Serous (n = 10)						**2**20%		**2**20%	**2**20%	**4**40%
	Endometrioid (n = 9)				**1**11%	**1**11%	**1**11%	**1**11%	**1**11%	**4**44%	
	Clear cell (n = 11)					**1**9%	**1**9%		**2**18%	**5**45%	**1**9%
	Mucinous (n = 6)		**3**50%	**1**17%	**1**17%					**1**17%	
Micro-array(a)(n = 99)	Normal (n = 4)										
	Serous (n = 41)		**1**2%	**4**10%		**3**7%	**10**24%		**4**10%	**19**46%	
	Endometrioid (n = 37)		**10**27%	**1**3%		**10**27%	**3**8%		**3**8%	**10**27%	
	Clear cell (n = 8)								**6**75%	**2**25%	
	Mucinous (n = 13)		**8**61%	**1**8%		**1**8%	**1**8%		**1**8%	**1**8%	

For each category of grade and stage, top number (bold font) corresponds to number of patients and bottom number to their corresponding percentage value.

(a) From publicly available dataset [35].

n.a – denotes not available.

### Cell Lines

Primary cultures of normal ovarian surface epithelium (NOSE) cells were derived, as previously described [29], from ovaries of three participants with no prior history of ovarian cancer, following profilactic oophorectomy at the CHUM Hôpital Notre-Dame and informed consent. EOC cell lines were established as previously described [29]–[31], and were derived from serous epithelial ovarian tumors (TOV81D, TOV2223, TOV1946) or ascites fluid (OV1946, OV866), from an endometrioid ovarian tumor (TOV112D), a clear cell carcinoma (TOV21G), and a mucinous epithelial ovarian cancer (TOV2444). Cells were cultured in OSE medium (Wisent Inc., St-Bruno, QC, Canada) containing 2.5 µg/ml amphotericin B and 50 µg/ml gentamicin (both from Invitrogen, Burlington, ON, Canada). Culture media was supplemented with 15% fetal bovine serum (FBS, Invitrogen) for the NOSE cultures and 10% FBS for the EOC cell lines.

### Quantitative real-time RT-PCR (Q-PCR)

Total RNA was extracted with Trizol reagent (Invitrogen) from frozen tumor samples or directly from cells grown to 80% confluency as described previously [32]. RNA quality was routinely monitored by agarose gel electrophoresis and with the 2100 Bioanalyzer, using the RNA 6000 Nano LabChip kit (Agilent Technologies, Waldbronn, Germany). The cDNA synthesis was performed according to the protocol of the QuantiTect Reverse Transcription kit (Qiagen Inc., Mississauga, ON, Canada) using 1 µg of total RNA. The obtained cDNA solution was diluted ten times and 5 µl aliquots were used in each Q-PCR reaction. Quantitative amplifications were obtained using the Platinum® SYBR® Green Q-PCR supermix UDG (Invitrogen) and the following pair of primers: 5′-AAGCCCTCCTCCTCCTTAACC-3′ and 5′-AGCCAGGGTAGCATCGAC-3′ for *HYAL1*, 5′-CGCGCTCTACCCTGCACTC-3′ and 5′-TGAATCCGGCCTCAGGTAGTT-3′ for ERα, 5′-TGGGCTTACTGACCAACCTG-3′ and 5′-CCTGATCATGGAGGGTCAAA-3′ for ERβ, 5′-AGAGTCCCTGGTGTGAAGCAA-3′ and 5′-GACAGCGCAGAAGTGAGCATC-3′ for progesterone receptor (PR), 5′-GCGCTGGCTCACCCCTACCT-3′ and 5′-GCCCCAGGGTGCAGAGATGTC-3′ for *ERK1*. The above mentioned ERα, ERβ and PR primer sequences were obtained from a previous publication [33]. *ERK1* was chosen as the control gene based on its stable expression in ovarian samples, consisting of normal and different EOC subtypes [34], [35], and as previously described [34]. Fluorescence was captured using the iCycler iQ real-time detection system (BioRad Laboratories, Hercules, CA). Amplifications were carried out at 50°C for 2 minutes (UDG incubation), 95°C for 3 minutes (denaturing), and 50 cycles of 95°C/30 sec, 58°C/30 sec and 72°C/45 sec, followed by a melting curve of 70 cycles of 0.5°C increase/cycle starting at 60°C. Positive and negative controls were introduced in all experiments, and purity and specificity of the PCR products were sporadically monitored by agarose gel electrophoresis. PCR reactions were performed at least three times for each cDNA sample in separate experiments. Relative expression value was obtained by the 1/2^ΔCt^ method using *ERK1* as the control gene. In this method, the difference in C_T_ (ΔC_T_) between the target and the control genes was used to determine gene expression with the formula 2^ΔCT^. A normalized value of expression for the target gene with reference to the control gene was then obtained by the calculation 1/2^ΔCT^. All mRNA expression values were ratios relative to the control *ERK1* gene.

To monitor the biological response of ERα ectopic expression on TOV21G cells (see below), Q-PCR was performed to measure mRNA levels of known ERα targets, e.g. *GREB1* (growth regulation by estrogen in breast cancer 1) and *TFF1* (trefoil factor 1, also known as pS2 gene) [36], [37]. PCR reactions were performed as above described using the following pairs of primers: 5′-TTCCCCGAAGTGCCAACAAC-3′ and 5′-ATGGAGATTCTGGAGACCACCC-3′ for *GREB1*, and 5′-TGGAGAACAAGGTGATCTGCG-3′ and 5′-CGAAACAGCAGCCCTTATTTGC-3′ for *TFF1*.

### GEO Dataset Analysis

Gene expression profiles of several tissue samples of the four morphologically distinct ovarian cancers and of normal ovaries has been performed using the Affymetrix HG_U133A array [35] and was made accessible through the National Center for Biotechnology Information (NCBI) (GEO dataset GSE6008). In the present study, we uploaded the raw table for each tumor sample and selected the normalized values [quantile-normalized trimmed-mean, log-transformed with log(max(x+50,0)+50] for the hybridization of the probes of interest. In the case of *HYAL1* and PR, the Affymetrix HG_U133A array contained only one probe for each of these genes (210619_s_at and 208305_at, respectively). Therefore, these values were used as such in our analysis to evaluate the expression of these genes in individual tissue samples. However, for ERα and ERβ several probes were available (205225_at, 211233_x_at, 211234_x_at, 211235_s_at, 211627_x_at, 215551_at, 215552_s_at, 217163_at, 217190_x_at for ERα; and 210780_at, 211117_x_at, 211118_x_at, 211119_at, 211120_x_at for ERβ), and the average of the normalized values for the probes of each gene was used in our work to analyse the expression of these genes in individual tissue samples. Information concerning histology, grade and stage of these samples was obtained from the available supplemental material [35] and is summarized in Table 1.

### Hyaluronan zymography

To monitor hyaluronidase activity of cell lysates from ovarian cancer cell lines, hyaluronan zymography was performed as previously described [38]. Cells harvested from 80% confluent cultures were resuspended in lysis buffer (10 mM imidazole, 0.25 M sucrose), briefly sonicated and their protein content determined by the BioRad Protein Reagent. Samples (containing 30 µg of protein) were separated by native PAGE (20 mA, 4°C) on an 8% gel containing 0.25 mg/ml human umbilical cord hyaluronic acid (Sigma-Aldrich, Oakville, Ontario, Canada). The gel was then briefly equilibrated in the assay buffer specific for hyaluronidase-1 detection (100 mM sodium formate, 150 mM NaCl, pH 3.7) and subsequently incubated overnight in the same buffer at 37°C. After incubation, gels were treated with 0.01 mg/ml Pronase (Sigma-Aldrich) in a 20 mM Tris buffer (pH 8.0) for 4 h, rinsed with distilled water, and stained sequentially with 0.5% Alcian blue and 0.1% Coomassie blue R, both in 30% methanol∶10% acetic acid. Gels were de-stained until clearing bands of digested hyaluronan were evident. Gel zymography was repeated three times for each cell line using cell extracts from different cultures.

### HYAL-1 enzymatic activity of plasma samples

Quantification of HYAL-1 activity in plasma sample was assayed by a colorimetric method for the estimation of *N*-Acetyl-D-glucosamine (NADG) released after hyaluronan digestion [39], [40]. Briefly, aliquots of EDTA-treated plasma (around 4 µl, containing 30 µg proteins, as determined by the BioRad Protein Reagent) were incubated with 40 µg of hyaluronan from human umbilical cord (Sigma-Aldrich) in 200 µl final volume of reaction buffer (79 mM sodium formate, 150 mM NaCl, 0.2 mg/ml BSA, pH 3.9) at 37°C for 24 h. The reaction was terminated by addition of 40 µl of 1.2 M potassium tetraborate pH 9.1 and boiled for 3 min. Colour formation is revealed by the addition of 1.2 ml of *p*-dimethylaminobenzaldehyde reagent (prepared as previously described [39], [40]) and incubation at 37°C for 20 min in the acidic environment of this reagent. Samples were immediately read at 585 nm. Hyaluronidase activity was estimated from a NADG (Sigma-Aldrich) standard curve (1 to 10 nmols), and was expressed as nmol/µg protein. Plasma protein content was determined by the BioRad Protein Assay. Blanks were obtained by omitting the plasma samples. Each plasma sample was measured at least three times in separate experiments.

### TOV21G cell transfection

pCDNA plasmid (Invitrogen) containing the human estrogen receptor alpha sequence (named pERα) has been described [41], [42]. TOV21G cells were cultured in complete OSE (Wisent) medium as described above until cells were around 60% confluent. Cells were transiently transfected with 1 µg pERα in DMEM-F12 medium (Wisent) without supplements using the GeneJuice transfection reagent (EMD Chemicals, Gibbstown, NJ), according to the manufacturer's instruction. After an overnight transfection, the medium was replaced by complete OSE medium, and cells were cultured for an extra 24 h for protein expression. At the end of the experiment, cells were harvested by trypsin-EDTA treatment (0.25% trypsin with 1 mm EDTA; Invitrogen), washed in PBS and used for RNA extraction and Q-PCR or for protein extraction and immunoblotting. Transfection experiments were repeated at least three times.

### Immunoblotting

Cells were lysed in Tris-buffered saline (20 mM Tris-HCl, 150 mm NaCl, pH 7.4)(TBS) containing 0.1% Triton X-100, 1 mM orthovanadate, 1 mM NaF, 0.1 mM PMSF and 1× protease inhibitor cocktail (Complete-mini, Roche Diagnostics Canada, Laval, QC, Canada). Protein content was determined by the BioRad Protein Reagent using a BSA standard curve. Samples (lysates of approximately 30 µg protein) were subjected to 12% SDS-PAGE, under reducing conditions, and electrotransferred onto nitrocellulose membranes. Non-specific binding to the membrane was blocked with 5% dehydrated skim milk in TBS. Membranes were then incubated with primary antibodies (4°C, overnight), washed in TBS containing 0.1% Tween, and incubated with horseradish peroxidase conjugated secondary antibodies. The primary IgG antibodies used in our work were anti-ERα (H-184, 1∶1000, rabbit antibody; Santa Cruz Biotechnology, Santa Cruz, CA) and anti-actin (pan Ab-5, 1∶1500, mouse antibody; Lab Vision Corp., Fremont, CA). These antibodies were tested in our conditions to be specific for their target proteins. Peroxidase-conjugated anti-mouse IgG (1∶1000, goat antibody; Sigma) and anti-rabbit IgG (1∶3000, goat antibody; Bio-Rad) were used as secondary antibodies. Protein-antibody recognition was detected by Western Lightning Chemiluminescence Reagent Plus (PerkinElmer, Boston, MA), according to the manufacturer's instructions.

### Statistical analysis

Because the levels of HYAL-1 and hormone receptors were not always normally distributed, nonparametric tests were used to analyse gene expression in tissue samples and enzymatic activity in plasma samples. We first performed a nonparametric Kruskal-Wallis analysis of variance and when significant differences were observed, we conducted Mann Whitney tests comparing the group of normal (for microarray) or benign (for Q-PCR and activity) samples with each of the tumor subtypes separately. Statistical significance was considered at *P*<0.05. Spearman's rank correlation coefficients were calculated and were considered statistically significant when *P*<0.05. Differences in gene expression of transfected and non-transfected TOV21G cells were analysed by the Student's *t*-test (two-tailed, equal-variance) and were considered significant when *P*<0.05. All statistical analyses were done using Prism4 for Windows version 4 (GraphPad Software, Inc.).

## Results

### Hyaluronidase-1 expression is elevated in clear cell and mucinous but not in serous and endometrioid EOC

**Figure 1:**
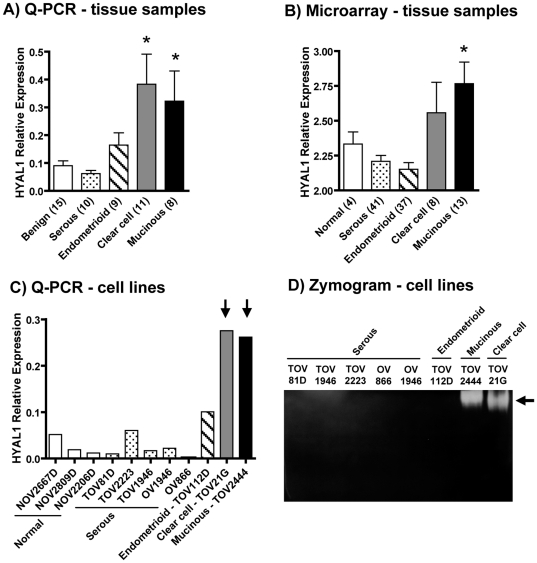
HYAL-1 expression in ovarian cancer tissue samples and cell lines. A) Q-PCR for *HYAL1* mRNA expression in benign tumor (white bar), and in serous (dotted bar), endometrioid (hatched bar), clear cell (shaded bar) and mucinous (black bar) EOC tissue samples from patients. Bars represent the mean ± SEM of the relative *HYAL1* expression normalized to control gene *ERK1* from different patients in each group. The value for each individual cDNA sample is the mean of 3–4 separate Q-PCR measurements. * denotes *P*<0.05 on Mann Whitney test. B) Normalized values for *HYAL1* probe hybridization of the Affymetrix HG_U133A array (GEO dataset GSE6008) on RNA samples of normal ovarian tissues (white bar), and of serous (dotted bar), endometrioid (hatched bar), clear cell (shaded bar) and mucinous (black bar) EOCs. Bars represent the mean ± SEM of raw values of normalized *HYAL1* hybridization. * denotes *P*<0.05 on Mann Whitney test. C) Q-PCR for *HYAL1* mRNA expression in cell lines derived from normal ovarian epithelium (NOV2667D, NOV2809D, NOV2206D; white bars), and from serous (TOV81D, TOV2223, TOV1946, OV1946, OV866; dotted bars), endometrioid (TOV112D; hatched bar), clear cell (TOV21G; shaded bar) and mucinous (TOV2444; black bar) EOCs. Bars represent the mean of the relative *HYAL1* expression normalized to control gene *ERK1* for each cell line. Q-PCR measurements were repeated at least three times for each cell line cDNA. Cell lines derived from different EOC subtypes are represented in separate bars therefore no error bars are represented. Vertical arrows indicate clear cell and mucinous cell lines having high *HYAL1* mRNA expression. D) Hyaluronan zymogram of cell lysates from the above mentioned serous, endometrioid, clear cell and mucinous ovarian cancer cell lines. The horizontal arrow marks the clear band of digested hyaluronan. The image is representative of three independent zymogram experiments.

In the past, the expression of Hyaluronidase-1 in ovarian cancer has been described specifically in serous tumors [16], [17], most probably because this is the most frequent EOC subtype [4]. In the present study we analysed the mRNA expression of this enzyme by Q-PCR in ovarian cancer tissue samples obtained from patients with different morphological subtypes of this disease, e.g. serous (11 samples), endometrioid (9 samples), clear cell (11 samples) and mucinous (8 samples). Expression levels were then compared to those of 15 benign ovarian tumors. Benign tumors were chosen for comparison due to their abundant content of surface epithelium which is very few in normal ovaries. In addition, we wanted to avoid possible interference of HYAL-1 expression in other ovarian cell types. For instance, we have shown that this enzyme is expressed in mouse ovarian granulosa cells [38]. As shown in Figure 1A, levels of *HYAL1* mRNA are significantly elevated in clear cell and mucinous carcinomas (Mann Whitney test, *P*<0.05) but not in serous and endometrioid tumors.

Because the size of our sample cohort could be considered small, we decided to validate our results by analysing *HYAL1* mRNA expression in a publicly available microarray dataset [35] (GEO dataset GSE6008) containing 41 serous, 37 endometrioid, 8 clear cell and 13 mucinous EOC samples, and 4 normal ovarian tissue samples. Figure 1B shows the normalized values [quantile-normalized trimmed-mean, log-transformed with log(max(x+50,0)+50] for the hybridization of each sample with the *HYAL1* probe (210619_s_at) of the Affymetrix HG_U133A array. Consistent with our Q-PCR results, no significant difference was observed for *HYAL1* mRNA expression between normal ovarian tissues and that of serous or endometrioid tumor samples (Figure 1B). Clear cell carcinomas had a tendency to express higher levels of *HYAL1* mRNA than normal ovarian tissue but no statistical significance was attained. In accordance to our results, mucinous samples had statistically significant higher levels of *HYAL1* than normal tissue (Mann Whitney test, *P*<0.05) (Figure 1B).

Ovarian cancer cell lines derived from tumor samples of these different morphological subtypes have been previously characterized and showed behaviour similar to the tumor sample from which they originated [29]–[31]. They provide powerful tools to investigate the molecular events related to each morphologically distinct ovarian cancer. Therefore, our next step was to analyse the level of expression of *HYAL1* mRNA and protein in these cell lines. Primary cell cultures of normal ovarian surface epithelium (NOSE) [29] were used as controls. Figure 1C shows that, as expected, mRNA expression of *HYAL1* were particularly high in cell lines TOV21G (derived from a clear cell carcinoma) and TOV2444 (derived from a mucinous EOC). An intermediate level of *HYAL1* mRNA expression was observed in the cell line TOV112D, which was derived from an endometrioid ovarian tumor. Cell lines derived from serous epithelial ovarian cancer at the primary site (TOV81D, TOV2223, TOV1946) or from the ascites fluid (OV1946, OV866) showed low levels of *HYAL1* expression, comparable to those of NOSE cell lines. In order to demonstrate that mRNA expression levels, measured quantitatively by our set of primers, reflected the amount of HYAL-1 protein in these samples, we measured HYAL-1 enzymatic activity by a substrate-gel zymogram. This is a conventional method to measure hyaluronidase activity and is specific for Hyaluronidase-1 when assayed at acidic pH [38]. Figure 1D shows a clear band of digested substrate (hyaluronan) only in the cell lysates of TOV21G and TOV2444 cell lines. The HYAL-1 enzymatic activity in the serous and endometrioid samples was below the detection level of our assay.

Because elevated HYAL-1 levels have been previously correlated with higher grade bladder and prostate tumors [21], [22], we decided to verify whether levels of *HYAL1* mRNA in clear cell and mucinous ovarian tumors would correlate with disease grade or stage (see Table 1 for patients' information). However, Spearman correlation analyses did not reveal a significant positive correlation between levels of *HYAL1* mRNA and either grade or stage in clear cell (r = 0.262 and −0.322 for grade and stage respectively in our data set, and r = 0.055 for stage in the microarray data, all *P*>0.05) or mucinous tumor samples (r = −0.655 and −0.577 for grade and stage respectively in our data set, and r = −0.094 and −0.378 for grade and stage in the microarray data, all *P*>0.05). Global analysis including all subtypes did not show a significant positive correlation either (r = −0.234 and −0.251 for grade and stage respectively in our data set, and r = 0.185 and 0.042 for grade and stage in the microarray data, all *P*>0.05). These results suggest that HYAL-1 expression might be an intrinsic phenotypic characteristic of clear cell and mucinous EOCs and that it might be used as diagnostic/detection marker for these ovarian cancer subtypes.

### Hyaluronidase-1 activity can be detected in the conditioned culture medium of TOV21G cell line and is a potential serum/plasma biomarker for clear cell and mucinous EOC

**Figure 2:**
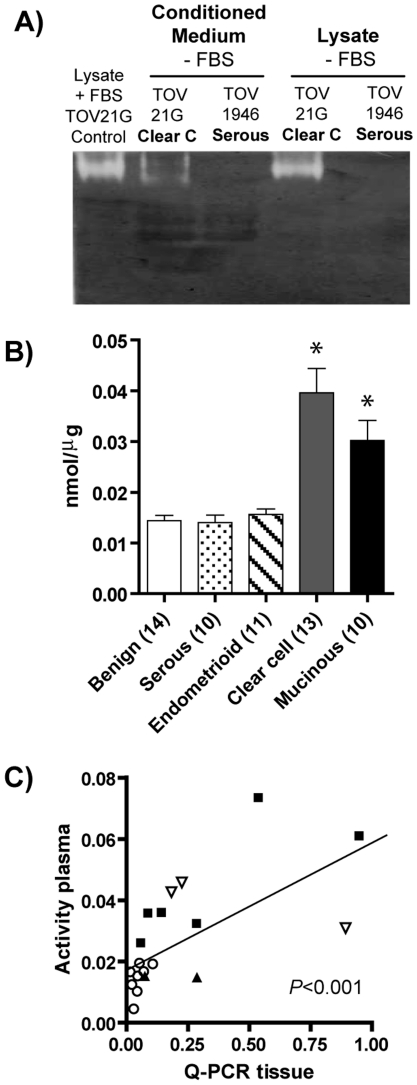
Secreted HYAL-1 protein as a potential plasma biomarker for the identification of clear cell and mucinous EOC. A) Hyaluronan zymogram of cell lysates (lanes 1, 4 and 5) and concentrated conditioned culture media (lanes 2 and 3) from serous (TOV1946) and clear cell (TOV21G) ovarian cancer cell lines. Note the clear band of digested hyaluronan specifically with the TOV21G samples. The image is representative of three independent experiments. B) HYAL-1 enzymatic activity of plasma samples were assayed quantitatively by a modified Reissig method [39], [40]. Bars represent the mean ± SEM of HYAL-1 activity measured in plasma samples of patients with benign ovarian tumor (white bar), or with serous (dotted bar), endometrioid (hatched bar), clear cell (shaded bar) or mucinous (black bar) EOCs. The value for each plasma sample is the mean of 3–4 measurements performed independently. * denotes *P*<0.05 on Mann Whitney test. C) Correlation graph of HYAL-1 enzymatic activity in plasma samples with *HYAL1* mRNA expression in tissue samples from patients with serous (white circles), endometrioid (black triangles), clear cell (black squares) and mucinous (white inverted triangles) EOCs. Each point represents the correlation value for one patient. Significance (*P*<0.01) was analysed by the Spearman correlation coefficient.

It has been shown that HYAL-1 is present in the culture medium of prostate and bladder cancer cell lines as well as in the urine of patients with bladder cancer [21], [26], [27]. In the present work, we wanted to verify whether HYAL-1 was also secreted by EOC cell lines and whether it could be used as a serum/plasma marker for the two morphological subtypes in which its expression level is elevated, e.g. clear cell and mucinous EOC. Figure 2A shows the presence of HYAL-1 activity (clear band of digested hyaluronan) in the concentrated serum-free conditioned medium of TOV21G cell culture as well as in its cell lysate. In contrast, no enzymatic activity can be detected in concentrated conditioned medium and cell lysate from the TOV1946 cell line (derived from a serous EOC). Having established that HYAL-1 is secreted by ovarian cancer cells expressing this enzyme, we investigated whether this enzymatic activity could be differentially detected in the plasma of patients with distinct morphological EOC subtypes. Because HYAL-1 has long been known to be present in mammalian serum/plasma [43], [44], a method other than the gel-substrate zymogram is needed to better determine differences in plasma/serum levels of this enzyme. For this purpose, we used a quantitative method for HYAL-1 activity measurement in solution [39], which has been recently used to detect the activity of this enzyme in human serum and plasma [40]. The activity of HYAL-1 was assayed in the plasma of patients with serous (10 samples), endometrioid (11 samples), clear cell (13 samples) or mucinous (10 samples) EOC, and compared to activities in plasma from 14 patients having benign ovarian tumors. As expected, HYAL-1 enzyme activity was detected in all plasma samples (Figure 2B). However, levels of this enzyme were significantly higher in plasma samples from patients with clear cell (0.0396±0.0131 nmol/µg, *P*<0.0001) and mucinous (0.0302±0.0107 nmol/µg, *P*<0.005) EOC than those of patients with benign tumors (0.0144±0.0032 nmol/µg) (2.8 and 2.1 fold increase respectively). HYAL-1 levels in plasma from patients having serous or endometrioid EOC (0.0140±0.0036 and 0.0157±0.0025 nmol/µg respectively) were similar to those of benign.

For some EOC patients we had paired analysis of *HYAL1* Q-PCR from tissue samples and HYAL-1 activity from plasma samples. Importantly, Spearman correlation analysis revealed a significant correlation between levels of this enzyme in tumor tissue and circulating plasma (Figure 2C, r = 0.706, *P*<0.001), reinforcing our assumption that this enzyme can be potentially used as a biomarker for the detection of clear cell and mucinous EOCs.

### Expression of *HYAL1* in clear cell and mucinous EOC inversely correlates with those of ERα and PR

**Figure 3:**
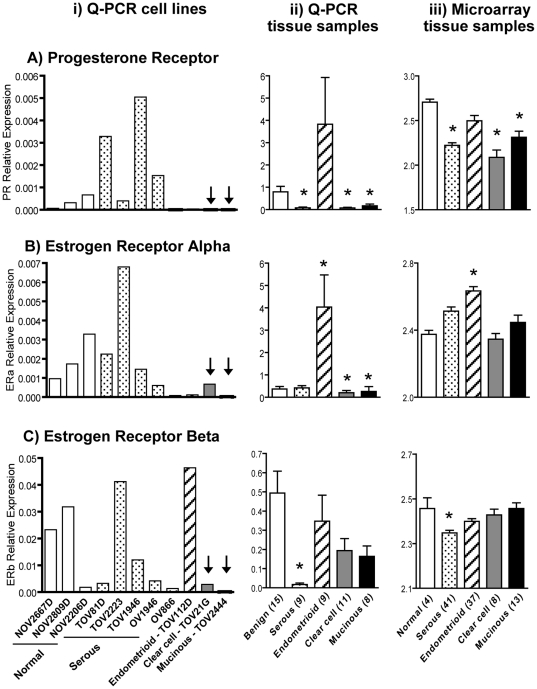
mRNA Expression of progesterone receptor (A), and estrogen receptors alpha (B) and beta (C) in ovarian cancer cell lines and tissue samples. i panels) Q-PCR for these receptors was performed on cDNAs from cell lines derived from normal ovarian epithelium, and from serous, endometrioid, clear cell and mucinous EOCs (see Figure 1C legend for details). Bars represent the mean of the relative mRNA expression for each receptor normalized to the control gene *ERK1*. Q-PCR measurements were repeated 3–4 times for each receptor and each cell line. As for Fig. 1, cell lines derived from different EOC subtypes are represented in separate bars therefore no error bars are represented. Vertical arrows indicate clear cell and mucinous cell lines having low mRNA expression of these receptors. ii panels) Normalized mRNA expression levels for steroid receptors determined by Q-PCR on cDNAs from benign tumors, and from serous, endometrioid, clear cell and mucinous EOC tissue samples (see Figure 1A legend for details). Q-PCR measurements were performed at least three times for each receptor and each tissue sample, and bars represent the mean ± SEM of the relative mRNA expression for each receptor normalized to the control gene *ERK1* in each tissue category. * denotes *P*<0.05 on Mann Whitney test. iii panels) Normalized values for hybridization of PR, ERα and ERβ probes of the Affymetrix HG_U133A array (see Material and Methods for details) on RNA samples of normal ovarian tissues, and of serous, endometrioid, clear cell and mucinous EOCs (see Figure 1B legend for details). Bars represent the mean ± SEM of the normalized raw values for the expression of each receptor in each tissue category. * denotes *P*<0.05 on Mann Whitney test.

Although the etiology of ovarian cancer is not clear, reproductive factors have been consistently recognized as risk factors. For instance, age at menopause and infertility contribute to a greater risk of ovarian cancer, whereas previous pregnancy, tubal ligation, and hysterectomy reduce risk. Oral contraceptive use has clearly been shown to be protective against ovarian cancer [45], [46]. In contrast, large epidemiologic studies found menopausal hormone replacement therapy to be a significant risk factor [47], [48]. Accordingly, and similarly to breast cancer, reports have shown that low ER and PR levels in ovarian cancers are associated with reduced survival and poor prognosis [49]–[53]. Interestingly, hyaluronidase activity has been shown to be elevated in ER-negative breast cancer cell lines [24]. Therefore in the present study we analysed the mRNA expression levels of ERα, ERβ and PR in the above mentioned ovarian cancer cell lines by Q-PCR. Our results show that mRNA expression of PR (Figure 3A, panel a), ERα (Figure 3B, panel a) and ERβ (Figure 3C, panel a) appears higher in cell lines possessing low *HYAL1* mRNA expression (Figure 1C) and vice-versa. In particular, TOV21G and TOV2444 cell lines have high levels of *HYAL1* mRNA expression and very low mRNA levels of all three receptors (see vertical arrows in both Figures 1 and 3). Our findings, using a panel of ovarian cancer cell lines, are concordant with those of breast cancer cell lines [24].

However, to our knowledge, this inverse relationship between Hyaluronidase-1 and hormone receptors has not been demonstrated in tissue samples from cancer patients. Therefore, we decided to address this issue in the present study. First, we characterized the level of mRNA expression of all three receptors (PR, ERα, ERβ) in our sample cohort by Q-PCR (Figure 3, b panels) and we examined the mRNA expression of these receptors in the publicly available microarray GEO dataset GSE6008 (Figure 3, c panels). Figure 3A shows that PR mRNA expression is significantly lower (*P*<0.05) in serous, clear cell and mucinous ovarian cancers when compared to benign tumors (panel b, our Q-PCR results) or to normal ovarian tissue (panel c, microarray results). ERα mRNA expression was also significantly lower (*P*<0.05) in clear cell and mucinous tumors although no significant reduction was observed in the microarray dataset (Figure 3B). However, ERα expression was significantly higher (*P*<0.05) in endometrioid ovarian carcinomas in both datasets (Figure 3B). For ERβ, figure 3C shows that its mRNA expression is significantly low only in serous epithelial ovarian cancer in both data analyses.

**Figure 4:**
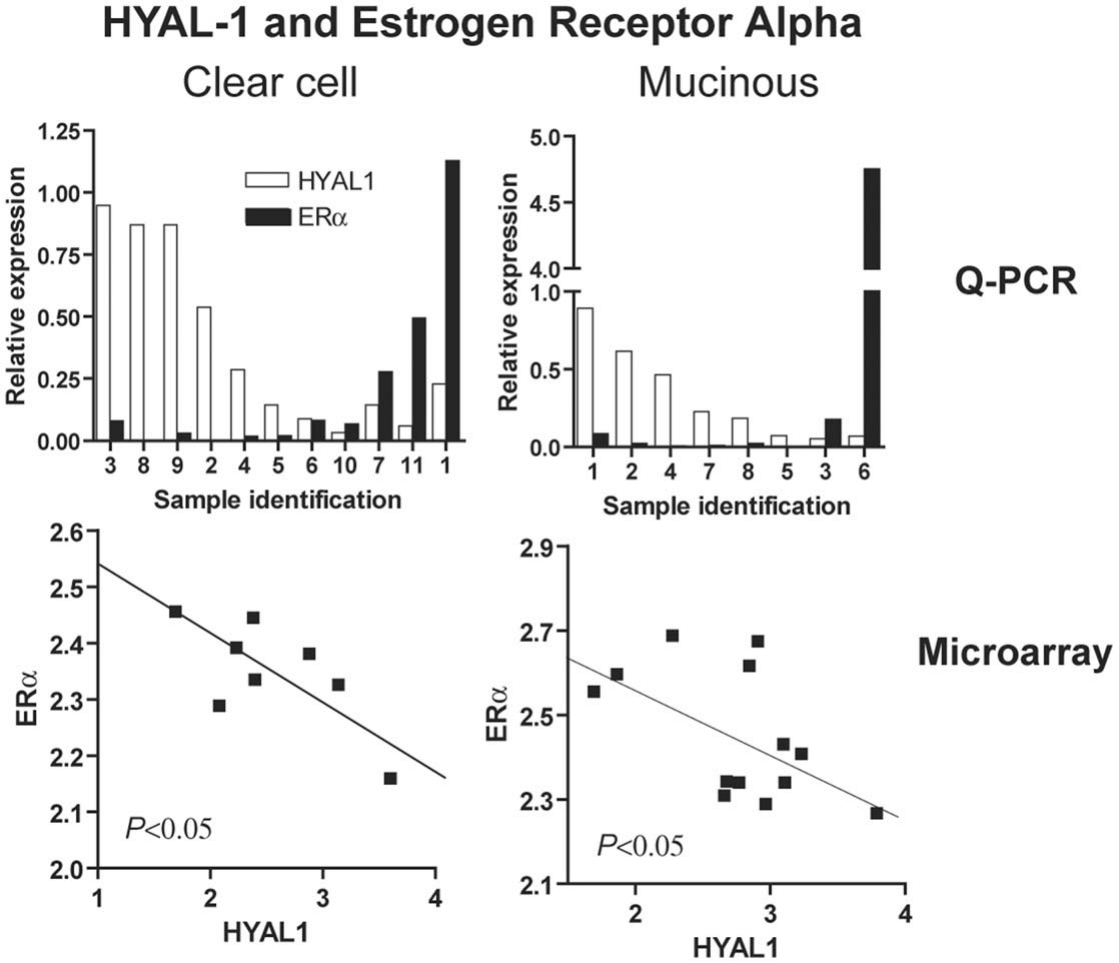
Inverse correlation of *HYAL1* expression with that of estrogen receptor alpha. In upper panels, bars represent relative mRNA levels of *HYAL1* (white bars) and ERα (black bars) measured by Q-PCR for each individual tissue sample. In lower panels, points represent relative mRNA expression values for each tissue sample from the microarray dataset (GSE6008). Left panels are correlations for clear cell tissue samples and right panels for mucinous tissue samples. Significance (*P*<0.05) was analysed by the Spearman correlation coefficient.

We further went on to verify whether expression of Hyaluronidase-1 inversely correlated with that of ERα, ERβ and/or PR in ovarian tumor samples. Spearman correlation coefficients (r) were calculated to examine a correlation between mRNA expression of *HYAL1* and steroid hormone receptors. Negative correlations were consistently observed between *HYAL1* mRNA expression levels and those of ERα and PR in clear cell and mucinous ovarian cancer subtypes (r values for *HYAL1* correlation with ERα in clear cell and mucinous EOCs were respectively −0.461 and −0.310 in the Q-PCR analysis and −0.595 and −0.467 in the microarray; r values for *HYAL1* correlations with PR in clear cell and mucinous EOCs were respectively −0.215 and −0.429 in the Q-PCR analysis and −0.500 and −0.352 in the microarray) (see bold fonts in Supplemental Table S1). This inverse correlation was statistically significant for ERα in clear cell and mucinous samples from the microarray dataset (Figure 4, lower panels, *P*<0.05). For the Q-PCR dataset, although no significance was obtained in the Spearman correlation for *HYAL1* and ERα levels in clear cell and mucinous EOCs, it is noteworthy that tissue samples exhibiting high *HYAL1* levels possess lower ERα expression and vice-versa (Figure 4, upper panels). Similar results were obtained with *HYAL1* and PR correlations in clear cell and mucinous EOCs (Supplemental Figure S1). For the other tested samples (Supplemental Table S1), correlation coefficients were not consistent between the Q-PCR and the microarray data.

To our knowledge this is the first indication demonstrating a negative correlation between *HYAL1* mRNA expression and ERα mRNA levels in epithelial ovarian cancer tumors.

### Regulation of the *HYAL1* gene by the estrogen receptor alpha in TOV21G cell line

**Figure 5:**
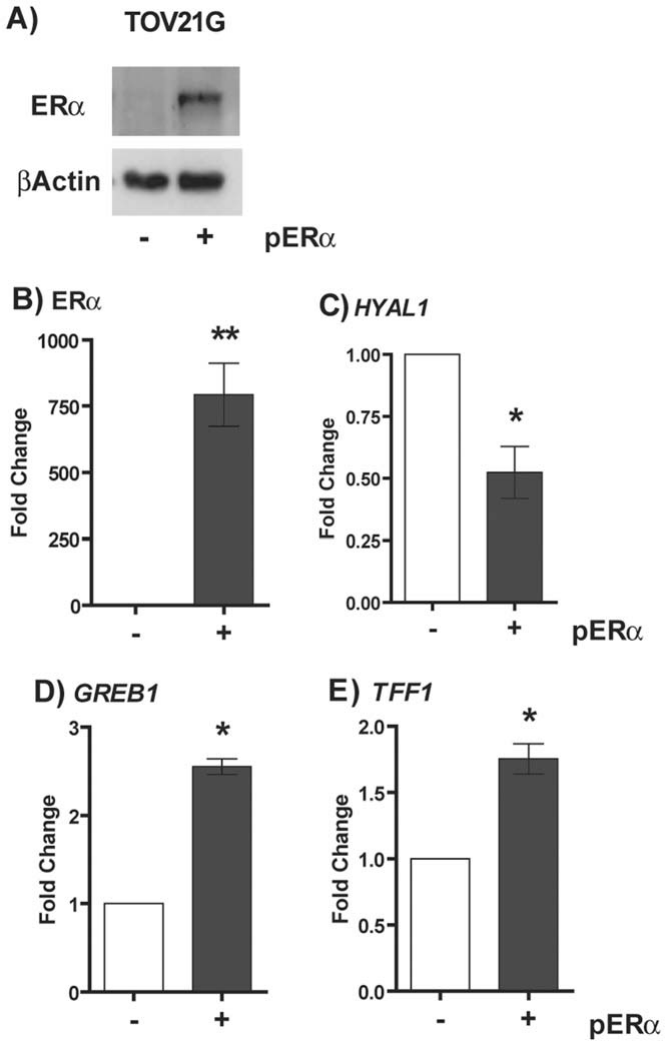
Ectopic expression of estrogen receptor alpha (ERα) negatively regulates *HYAL1* expression in TOV21G cell line. Plasmid containing the cDNA sequence of human ERα (named pERα) was transiently transfected in clear cell ovarian cancer cell line (TOV21G) and expression efficiency was analysed by immunoblotting (A) and Q-PCR (B). mRNA levels of *HYAL1* (C), *GREB1* (D) and *TFF1* (E) were also analysed by Q-PCR in these transfected cell lines. (−) denotes control cells transfected with empty plasmid, and (+) denotes pERα transfected cells. In A, immunoblotting was performed using anti-ERα antibody and the anti-βActin antibody was used as control.

To determine whether *HYAL1* gene is under direct regulation by ERα, we ectopically express ERα in ovarian TOV21G cells which are derived from clear cell EOC, are negative for ER and PR (Figure 3) and express high HYAL-1 levels (Figure 1). Figures 5A and 5B demonstrate that ERα was efficiently expressed in this cell line showing high mRNA and protein levels after transient transfection. Functional activity of ERα was confirmed by a significant activation of known ER target genes [36], [37] such as *GREB1* (2.55 fold increase in expression, *P*<0.01) and *pS2/TFF1* (1.75 fold increase in expression, *P*<0.05) (Figures 5D and 5E). Using such approach, we observed that expression of ERα in TOV21G cells induced a 50% decrease in the *HYAL1* mRNA expression compared to mock-transfected cells (*P*<0.01), indicating that *HYAL1* gene expression is down-regulated by ERα (Figure 5C).

## Discussion

There are more than 190,000 new cases of epithelial ovarian cancer (EOC) each year worldwide and this malignancy represents the leading cause of death from gynaecological cancers [1], [2]. It is a complex disease, largely asymptomatic, and over 70% of patients present with advanced stage disease at initial diagnosis. EOC is a heterogeneous disease, and each EOC subtype exhibits distinct clinical characteristics, morphology, biological behaviour, and chemotherapeutic response [3]. Indeed, molecular studies support the notion that the different histological types likely represent distinct disease states [54], [55]. Nonetheless, it is current practice to treat all subtypes with the same platinum/taxane chemotherapy, although some do not respond well to this regimen [56]–[58]. As a consequence subtype-specific therapeutic trials have been recommended for clear cell and mucinous EOCs in particular [59]. The molecular and morphological differences of these EOC subtypes are also reflected in the efficiency for detection using the only approved serum biomarker for ovarian cancer, CA125; while 60–80% of patients with endometrioid and serous EOC show high levels of serum CA125, only 20–30% of patients with clear cell and mucinous are positive for this serum biomarker [60]. Therefore, further genetic and molecular characterizations are needed for these latter EOC subtypes in order to identify molecules with the potential to improve early detection.

In the present work, we show that *HYAL1* mRNA expression is elevated in clear cell and mucinous EOC tissue samples, but not in serous and endometrioid samples, normal ovaries or benign tumors (Fig. 1). Similar results were obtained by applying two different techniques on two separate tissue sample cohorts. Concordantly, *HYAL1* mRNA levels and enzymatic activity were elevated in EOC cell lines derived from clear cell and mucinous subtypes (Fig. 1). No extensive studies have closely investigated the expression of HYAL-1 in individual EOC subtypes. Others that have exclusively examined serous tumours have reported low *HYAL1* mRNA expression levels [16] and unchanged hyaluronidase activities in tissue extracts [16], [17]. Our results showing unchanged levels of *HYAL1* mRNA and plasma activity in serous EOC (Fig. 1) are in accordance with these latter findings. However, they differ from those showing low *HYAL1* mRNA levels [16]. This discrepancy is probably related to differences in experimental settings used in each study regarding quantitative real time RT-PCR. Nevertheless, the differential expression of HYAL-1 in individual EOC subtypes is probably related to their distinct molecular characteristics. As mentioned before, the *HYAL1* gene is located in a tumor suppressor locus at chromosome 3p and cytogenetic studies have revealed recurrent anomalies of chromosome 3 in ovarian cancer that include a non-random loss of all or regions of the 3p arm [5], [61], [62]. However, there are several other candidate tumor suppressor genes in this region, including *SEMA3B* and *SEMA3F* [63], [64] as well as *RASSF1* (RAS association domain family 1). In fact, inactivation of the latter has been implicated in the development of more than 40 types of sporadic human cancers [65], [66]. Therefore, it is possible that the hyaluronidases are not the main tumor suppressor genes in this region, but may instead be consequentially affected by default mechanisms in some tumor types. These events are certainly more frequent in serous EOC which presents higher frequency of chromosomal instability than the other subtypes [3], [67]. The concept of hyaluronidases as tumor suppressors seems appealing, as there is a vast literature demonstrating the role of its substrate, hyaluronan, in tumor progression, survival and metastasis [19], [68], [69]. However, the roles of hyaluronidases as tumor promoters have also been extensively reported [22], [70]. These studies showed that up-regulation of hyaluronidases can promote cell cycle activation, induce extracellular matrix degradation and formation of angiogenic hyaluronan fragments, and are associated with tumor growth, invasion, and recurrence in several cancer types [22], [70].

Our current results are in favour of these latter reports. It is possible that elevated HYAL-1 levels would also have a tumor promoter role in clear cell and mucinous EOCs, and future functional studies are warranted to test this interesting possibility. Significantly, we showed that HYAL-1 activity is particularly high in the plasma of patients with clear cell EOC (Fig. 2). This was the first evidence showing high circulating HYAL-1 levels in ovarian cancer. It has long been known that HYAL-1 is present in biological fluids such as plasma and urine [44], [71]. Interestingly, levels of hyaluronan and HYAL-1 are reported to be elevated in urine of patients with bladder cancers [21], [22] and HYAL-1 has been shown to be secreted to the culture medium by some bladder and prostate cancer cell lines [26], [27]. Furthermore, patients with head and neck carcinomas are shown to have elevated hyaluronidase activity in their saliva [20]. Our results show that HYAL-1 is also secreted by the ovarian cancer cell line originating from the clear cell carcinoma, but not from that of serous EOC (Fig. 2). Although several molecular markers for EOC have been identified [3], [60], [67], [72], [73], CA125 remains the only clinically approved serum biomarker for the detection of this disease. However, as above mentioned, clear cell and mucinous EOCs are rarely detected by this assay. One interesting perspective would be to determine the efficacy and feasibility of a combined test to measure HYAL-1 plasma activity in conjunction with CA125 measurements for ovarian cancer screening.

Previous reports have shown that increased expression of hyaluronan and HYAL-1 were associated with high grade bladder and prostate cancers [21], [22]. However, in the present work, significant correlation between *HYAL1* mRNA levels and tumor grade or stage was not obtained for any EOC subtype. It is possible that *HYAL1* levels are associated with parameters other than disease grade or stage in clear cell and mucinous EOC. Our results rather indicate that HYAL-1 expression in EOC might reflect the intrinsic phenotypic characteristics of the distinct histological subtypes.

EOC is significantly more prevalent and severe in post-menopausal women. Throughout menopause, changes in the hormonal milieu ostensibly provide important regulatory switches for ovarian tumor progression. These changes include elevated levels of luteinizing hormone (LH) and follicular-stimulating hormone (FSH), and reduced levels of estrogen and progesterone. Accordingly, status of gonadotropin and steroid hormone receptors has been associated with ovarian cancer patients' survival [49]–[53], [74]–[76]. FSH and LH receptors are positively correlated with tumor progression and poor prognosis [74]–[76], as well absence of ER and PR has been associated with reduced survival and poor prognosis [49]–[53]. Interestingly, different EOC subtypes have distinct patterns of expression for specific steroid hormone receptors. We and others have shown that endometrioid EOCs are generally ER positive whereas clear cells are negative ([77] and Fig. 3). It is believed that this characteristic might account for the less aggressive phenotype of the endometrioid subtype [3]. Absence of ER in breast cancer has been consistently associated with tumor progression, recurrence and metastasis, and despite major advances in surgery and adjuvant therapies, ER-negative breast cancer remains difficult to treat [52], [78]. Interestingly, ER-negative breast cancer cell lines have higher hyaluronidase levels than ER-positive cells [24] and silencing of *HYAL1* by RNA interference inhibited cell proliferation and induced cell cycle arrest in some breast cancer cell lines [79]. Results from the present work demonstrate that ovarian cancer cell lines expressing high HYAL-1 levels, e.g. derived from clear cell and mucinous EOCs (Fig. 1), have low mRNA expression of ERα, ERβ and PR (Fig. 3). More importantly, we determined that *HYAL1* mRNA levels are inversely correlated with those of ERα specifically in clear cell and mucinous EOC tissue samples (Fig. 4), suggesting a role for ERα in regulating *HYAL1* gene expression in ovarian cancer. Previous studies have shown that CD44-hyaluronan interaction engages a cascade of signalling events to promote ERα activation in a serous ovarian cancer cell line [80]. However, to our knowledge this is the first demonstration of a correlation between *HYAL1* and ERα mRNA levels in ovarian tumor cells and more importantly in tumor samples collected from patients. Consistent with such regulation, we demonstrate that ectopic expression of ERα in a clear cell EOC cell line (negative for this ER receptor) induced a significant decrease in *HYAL1* gene expression (Fig. 5), which identifies *HYAL1* as an ER target for gene repression. We therefore postulate that regulation of *HYAL1* expression by ERα participates in ovarian cancer progression. As such HYAL-1 might play a role in tumor proliferation and cell cycle progression in ER-negative clear cell and mucinous EOC much in the same way as has been shown for ER-negative breast cancer [24], [79] and for bladder and prostate cancer cell lines [26], [27]. Therefore, it will be important to further understand the mechanism behind *HYAL1* gene regulation by ERα at the promoter level, and we are presently working on this subject.

In conclusion, we demonstrate that HYAL-1 is differentially expressed in morphologically distinct EOC subtypes, and that such regulation might implicate the estrogen receptor alpha. Our results also identify Hyaluronidase-1 as a potential target/biomarker for clear cell and mucinous EOCs and especially in tumors with low ERα levels.

## Supporting Information

Figure S1 Inverse correlation of *HYAL1* expression with that of progesterone receptor. In upper panels, bars represent relative mRNA levels of *HYAL1* (white bars) and PR (shaded bars) measured by Q-PCR for each individual tissue sample. In lower panels, points represent relative mRNA expression values for each tissue sample from the microarray dataset (GSE6008). Left panels are correlations for clear cell tissue samples and right panels for mucinous tissue samples.
(TIF)

Table S1 Correlation coefficients for *HYAL-1* mRNA expression with those of progesterone receptor (PR), or estrogen receptors alpha (ERα) or beta (ERβ).
(DOC)
