# Supplementary figures and images for: Subtype Specific Elevated Expression of Hyaluronidase-1 (HYAL-1) in Epithelial Ovarian Cancer
Source: PLoS One. 2011 Jun 10;6(6):e20705. doi: 10.1371/journal.pone.0020705 (PMC3112150; doi:10.1371/journal.pone.0020705)

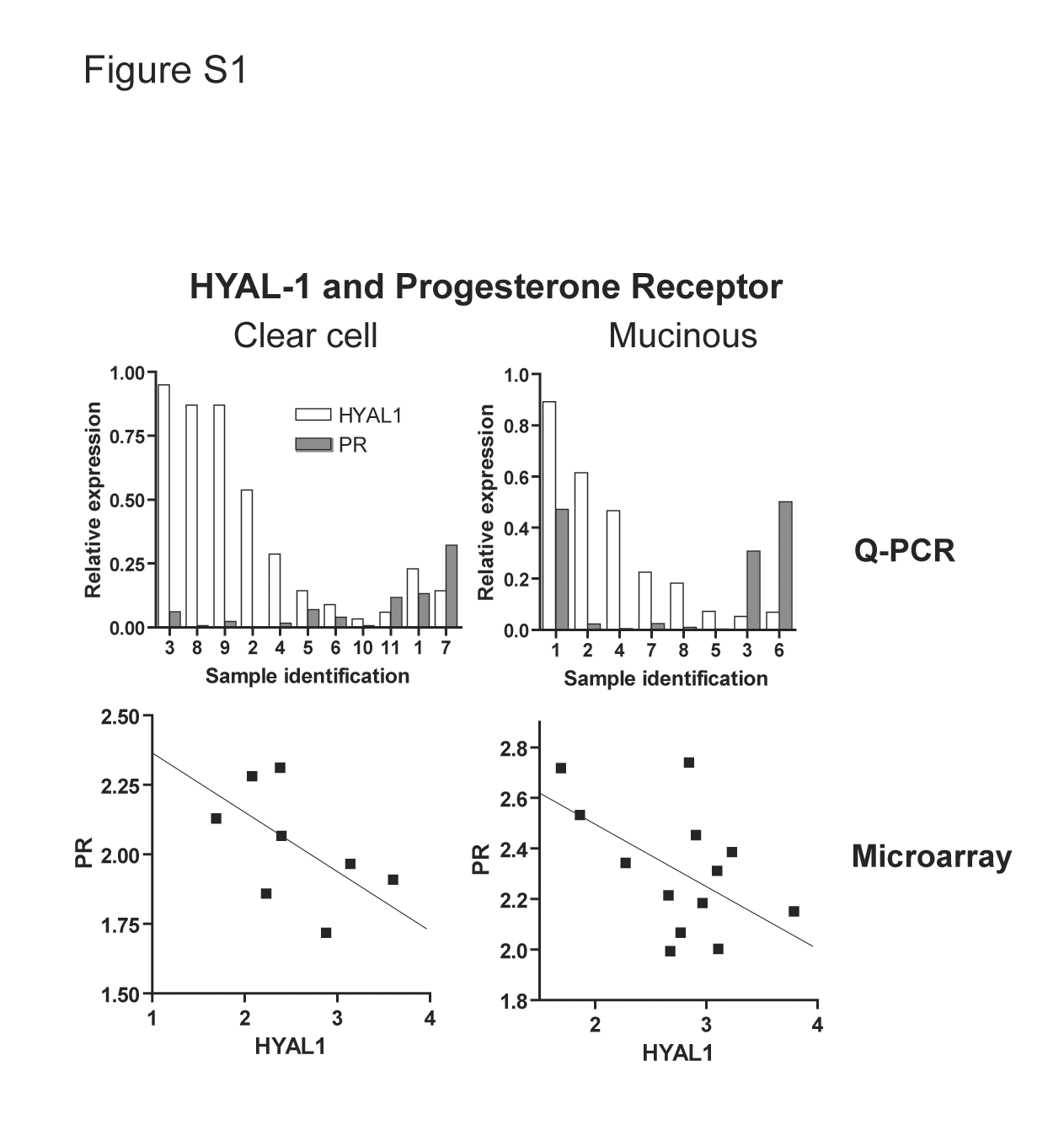

Supplement: Figure S1 — Inverse correlation of HYAL1 expression with that of progesterone receptor. In upper panels, bars represent relative mRNA levels of HYAL1 (white bars) and PR (shaded bars) measured by Q-PCR for each individual tissue sample. In lower panels, points represent relative mRNA expression values for each tissue sample from the microarray dataset (GSE6008). Left panels are correlations for clear cell tissue samples and right panels for mucinous tissue samples. (TIF) [file pone.0020705.s001.tif]
