# Supplementary material for: Subtype Specific Elevated Expression of Hyaluronidase-1 (HYAL-1) in Epithelial Ovarian Cancer
Source: PLoS One. 2011 Jun 10;6(6):e20705. doi: 10.1371/journal.pone.0020705 (PMC3112150; doi:10.1371/journal.pone.0020705)
Supplement: Table S1 — Correlation coefficients for HYAL-1 mRNA expression with those of progesterone receptor (PR), or estrogen receptors alpha (ERα) or beta (ERβ). (DOC) [file pone.0020705.s002.doc]

**Table S1** - Correlation coefficients for *HYAL-1* mRNA expression with those of progesterone receptor (PR), or estrogen receptors alpha (ERα) or beta (ERβ)

|  | | *Spearman r value for HYAL-1 expression correlation* | | | |
| --- | --- | --- | --- | --- | --- |
|  |  | *Serous* | *Endometrioid* | *Clear cell* | *Mucinous* |
| PR | Q-PCR | 0.309 | 0.817* | **- 0.215** | **- 0.429** |
|  | Microarray | 0.224 | - 0.263 | **- 0.500** | **- 0.352** |
| ERα | Q-PCR | 0.067 | 0.717* | **- 0.461** | **- 0.310** |
|  | Microarray | 0.149 | - 0.154 | **- 0.595*** | **- 0.467*** |
| ERβ | Q-PCR | 0.667 | 0.683 | 0.443 | - 0.119 |
|  | Microarray | 0.050 | - 0.411* | 0.214 | 0.637* |

* denotes *P*<0.05
